# Exploring the Role of Sleep and Physical Activity in Academic Stress, Motivation, Self-Efficacy, and Dropout Intention Among Italian University Students

**DOI:** 10.3390/ejihpe16010003

**Published:** 2025-12-22

**Authors:** Jessica Dagani, Chiara Buizza, Alberto Ghilardi

**Affiliations:** Department of Clinical and Experimental Sciences, Section of Clinical and Dynamic Psychology, University of Brescia, Viale Europa 11, 25123 Brescia, Italy; chiara.buizza@unibs.it (C.B.); alberto.ghilardi@unibs.it (A.G.)

**Keywords:** sleep, physical activity, university adjustment, students, well-being

## Abstract

University years represent a period of major transition during which health-related behaviors, such as sleep and physical activity, may influence students’ academic functioning. This cross-sectional, single-center study, conducted at an Italian university, examined the associations between sleep, physical activity, and academic well-being. Students completed an online survey assessing sleep, physical activity, and several indicators of academic functioning (i.e., academic stress, self-efficacy, dropout intention, and motivation). Nonparametric tests (Kruskal–Wallis, Jonckheere–Terpstra) were used to explore differences in these indicators across sleep quality and physical activity categories, while linear regressions tested associations between sleep duration and Metabolic Equivalent of Task–minutes/week with the same academic outcomes. A total of 2192 students (15.55%) accessed the survey, and 1246 (8.84%) completed all questionnaires. Most participants were female (62.7%) and Italian (94.5%). Both sleep and physical activity showed significant but small associations with academic stress, dropout intention, and self-efficacy, whereas associations with academic motivation were weaker. These findings suggest that maintaining regular physical activity and healthy sleep habits may contribute to students’ academic adjustment, although the cross-sectional design limits causal interpretation and underscores the need for integrative models to better understand the underlying psychological mechanisms.

## 1. Introduction

University years represent a period of major transition for students, encompassing separation from family, adaptation to new living arrangements, the establishment of new social relationships, and the management of increased academic demands. These changes occur during emerging adulthood, a particularly sensitive developmental stage characterized by profound psychological and social challenges. Taken together, these factors increase university students’ vulnerability to mental health problems and psychological difficulties (Auerbach et al., 2016). According to the Eurostudent 8 report, between 37% and 58% of European university students report poor well-being (Cuppen et al., 2024). Similar trends have been observed in Italy, where recent studies highlight elevated levels of academic pressure and psychological distress among university students (Bersia et al., 2024; Porru et al., 2021). Poor psychological well-being is also associated with reduced concentration and academic engagement, which may compromise academic performance and negatively affect future professional and personal development (Bruffaerts et al., 2018; Diaz Mujica et al., 2019).

In this context, increasing attention has been paid to health-related behaviors that may interact with psychological resources and influence students’ academic well-being. In the present study, we focus on two such behaviors, sleep patterns and physical activity levels, which have been identified as key contributors, with growing evidence supporting their potential role in sustaining academic performance (Armand et al., 2021; Li & Huang, 2025; Zhai et al., 2018). Sleep plays a fundamental role in memory consolidation, attention, emotional regulation, and executive functioning (Kong et al., 2023; Sen & Tai, 2023; Tomaso et al., 2021), all of which are essential for effective learning and engagement. However, university students frequently experience insufficient or poor-quality sleep due to academic stress, irregular schedules, and social demands (Becker et al., 2018). Sleep disturbances have been linked to depressive symptoms and suicidal ideation (Z. Wang et al., 2025; Yang et al., 2024), suggesting that poor sleep may impair not only students’ cognitive functioning but also their psychological resources. Similarly, physical activity exerts beneficial effects on cognition, mood, and stress regulation (Erickson et al., 2019; Singh et al., 2023). Among university students, low levels of physical activity are associated with poorer mental health outcomes (Grasdalsmoen et al., 2020; Rodríguez-Romo et al., 2023). Nevertheless, a significant proportion of students remain physically inactive, often prioritizing academic work or screen time over exercise (Castro et al., 2020). Both sleep and physical activity are increasingly recognized as crucial health-related behaviors with potential implications that extend beyond general well-being to academic functioning (Suardiaz-Muro et al., 2020; Haverkamp et al., 2020).

Among the various factors shaping academic well-being, four constructs have emerged as particularly relevant: dropout intention, academic stress, academic motivation, and academic self-efficacy. In this study, academic well-being is conceptualized as a multidimensional construct encompassing these four dimensions, which together reflect students’ ability to manage study-related demands, maintain engagement, and persist toward degree completion. These constructs are closely interconnected and play a central role in determining academic engagement and persistence. Dropout intention refers to students’ thoughts of leaving university before completing their degree, and empirical studies confirm its strong association with actual dropout behavior (Findeisen et al., 2024). Academic stress involves the perception of being overwhelmed by study-related demands, such as exams and deadlines, which can impair motivation, concentration, and cognitive functioning (Pascoe et al., 2019). Academic motivation, as conceptualized in Self-Determination Theory (Ryan & Deci, 2017), includes autonomous motivation (driven by intrinsic interest or personal values) and controlled motivation (driven by external or internal pressures). Autonomous motivation is typically associated with deeper engagement and better academic outcomes (Howard et al., 2021), whereas controlled motivation is more inconsistently linked to poorer performance (Santana-Monagas et al., 2025). Finally, academic self-efficacy, or students’ beliefs in their capacity to succeed, is a robust predictor of academic achievement (Fior et al., 2022).

Despite these findings, relatively few studies have examined how sleep and physical activity relate to specific dimensions of academic functioning and well-being (Chang et al., 2021; Jiang et al., 2025; Van de Casteele et al., 2025; Young-Jones et al., 2022). Existing research has produced mixed results, particularly regarding the associations between physical activity and academic stress, or between sleep quality and motivational regulation, highlighting the need for more integrative approaches. Moreover, most evidence derives from North American or Asian contexts, whereas research in Southern European countries remains scarce. Considering the cultural differences in sleep patterns, physical activity engagement, and academic demands across countries, examining these associations in an Italian sample may offer important insight into context-specific dynamics.

The present study aimed to investigate whether sleep quality and physical activity levels are associated with academic stress, academic self-efficacy, academic motivation, and dropout intention among Italian university students from a single university. Based on previous research, we hypothesized that sleep and physical activity would be negatively associated with academic stress and dropout intention, and positively associated with self-efficacy and motivation. These hypotheses align with prior findings showing that adequate sleep and regular physical activity predict lower stress and greater academic engagement (Herbert, 2022; Toscano-Hermoso et al., 2020). By focusing on Italian students, this research not only addresses a gap in the literature but also offers insights relevant for initiatives promoting student well-being in higher education. This is particularly important in Italy, where graduation rates remain comparatively low and early-year dropout has recently reached about 15% (Agenzia Nazionale di Valutazione del sistema Universitario e della Ricerca, 2023; Istituto Nazionale di Statistica, 2022).

**Figure 1:**
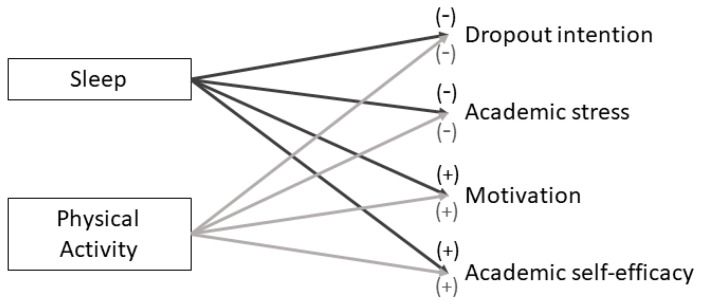
Conceptual model illustrating the hypothesized relationships between sleep, physical activity, and academic outcomes. Signs (+, −) indicate the expected direction of each association.

A conceptual model illustrating the hypothesized relationships between sleep, physical activity, and academic outcomes is presented in Figure 1. Unlike most previous studies focusing primarily on mental health outcomes, the present research places academic well-being at the center, clarifying how sleep and physical activity may support or hinder students’ engagement and persistence in higher education.

## 2. Materials and Methods

This cross-sectional observational study was conducted at a single medium-sized university located in Northern Italy. Data collection took place between November and December 2024. In collaboration with the University Secretariat, an institutional email invitation was sent to the entire student population. The invitation included a brief description of the study and a link to an online survey hosted on LimeSurvey (www.limesurvey.org, accessed on 18 July 2024), a secure platform ensuring anonymity of responses. Upon accessing the survey, students were presented with an informed consent form, which had to be accepted before proceeding. LimeSurvey automatically anonymized responses, ensuring that no identifying information was transmitted to the research team. The study received ethical approval from the Comitato Etico Territoriale Lombardia 6 (approval No. 0037967/24, 9 July 2024) and was conducted in accordance with the ethical principles of the Declaration of Helsinki. The study was reported following the STROBE (Strengthening the Reporting of Observational Studies in Epidemiology) guidelines for cross-sectional studies (see Supplementary Materials, Table S1).

### 2.1. Participants

The survey was distributed via an institutional email to the entire student population (14,093 students). A total of 2192 students (15.55%) accessed the survey, and 1246 (8.84%) completed all questionnaires. Promotion was intentionally minimal to ensure voluntary participation, which may have contributed to the moderate response rate.

A priori power analysis was not conducted due to the exploratory and cross-sectional nature of the study. Instead, the expected sample size was based on the eligible student population (approximately 14,000 enrolled students) and anticipated response rates from previous web-based surveys conducted at the same university (Dagani et al., 2025). Assuming a conservative 15% response rate, an expected sample of around 2,000 participants was considered sufficient to provide reliable estimates across study variables. The final sample exceeds empirical guidelines for multivariable analyses (e.g., Green, 1991; Peduzzi et al., 1996), which recommend at least 10–20 observations per predictor. Therefore, the sample size was considered adequate for the planned analyses. Effect sizes were also reported to provide an informative measure of association strength.

The majority of participants were female (62.7%) and Italian (94.5%), with 0.8% from other European countries and 4.7% from non-European countries. The mean age was 22.62 years (SD = 5.36). Most students were enrolled in Health Sciences (46.0%), Engineering and Agriculture (28.1%), or Economics programs (18.1%), while the remaining participants were enrolled in Law programs (7.8%). Participant characteristics are reported in Supplementary Table S2, including age, gender, nationality, field of study, and year of study. Year of study is reported for descriptive purposes only, given the heterogeneity of program lengths across disciplines.

### 2.2. Survey Instruments

The online survey collected information on socio-demographic characteristics, academic experience, and indicators of physical and mental well-being. The following standardized instruments were administered:

- University Stress Scale (USS, Stallman & Hurst, 2016). The USS is a 21-item measure assessing students’ cognitive appraisal of environmental stressors related to university life. Items are rated on a 4-point Likert scale ranging from 0 (“Not at all”) to 3 (“Constantly”). The total score, obtained by summing all items, ranges from 0 to 63. A score of 13 or higher indicate significant psychological distress. The scale was translated into Italian using a back-translation procedure, although formal validation of the Italian version was not conducted. In this study, Cronbach’s alpha value was 0.83.
- Academic Motivation Scale—adapted version (AMS, Biasi et al., 2017). Based on Self-Determination Theory (Vallerand et al., 1992), the adapted AMS assesses academic motivation across five subscales: Amotivation, External Regulation, Introjected Regulation, Identified Regulation, and Intrinsic Regulation. Each subscale includes four items rated on an 11-point Likert scale ranging from 0 (“Not at all true”) to 10 (“Completely true”). Subscale mean scores range from 0 to 10, with higher scores reflecting stronger endorsement of the corresponding motivational type. For the purposes of this study, two composite indices were computed (De Vincenzo, 2024): Controlled Motivation (combining External Regulation and Introjected Regulation) and Autonomous Motivation (combining Identified Regulation and Intrinsic Regulation). Controlled Motivation refers to engaging in academic activities due to external pressures or internal obligations, such as rewards, punishments, or feelings of guilt. In contrast, Autonomous Motivation reflects engaging in learning out of personal interest, enjoyment, or a sense of personal value and endorsement. Each composite score was calculated as the sum of the mean scores of the two corresponding subscales, resulting in a possible range from 0 to 20. In this study, Cronbach’s alpha values were 0.84 for Controlled Motivation and 0.93 for Autonomous Motivation.
- Perceived School Self-Efficacy Scale (SASP, Biasi et al., 2017; Pastorelli & Picconi, 2001). This 9-item scale measures students’ self-perceived ability to concentrate on and manage academic tasks. Items are rated on a 5-point Likert scale from 1 (“Not capable at all”) to 5 (“Fully capable”). The final score is computed as the mean of all items; higher scores indicate greater perceived academic self-efficacy. In the current sample, the scale demonstrated good internal consistency (Cronbach’s alpha = 0.89).
- Dropout Intention Scale—adapted version (Biasi et al., 2017; De Vincenzo, 2024; Hardre & Reeve, 2003). This 4-item measure assesses students’ intentions to withdraw from university. Items explore the frequency of thoughts and intentions related to dropping out. Each item is rated on a 5-point Likert scale ranging from 1 (“Never”) to 5 (“Always”). The final score is the mean of all items, with higher scores indicating stronger dropout intentions. In this sample, Cronbach’s alpha value was 0.93.
- International Physical Activity Questionnaire—Short Form (IPAQ-SF, Mannocci et al., 2010). The IPAQ-SF is a 7-item self-report instrument assessing physical activity over the past seven days. It captures the frequency and duration of walking, moderate-intensity (e.g., bicycling at a regular pace) and vigorous-intensity (e.g., heavy lifting) activities, as well as sedentary behavior. Total physical activity is quantified in Metabolic Equivalent of Task (MET–minutes/week), providing a standardized estimate of energy expenditure. In addition to this continuous measure, the IPAQ-SF also allows classification of individuals into three physical activity levels (low, moderate, high). While the IPAQ-SF may overestimate physical activity compared to objective measures (Lee et al., 2011), it remains one of the most widely used and validated instruments for epidemiological studies.
- Sleep was assessed using two self-report items: participants reported (a) their average number of hours of sleep per night and (b) their perceived sleep quality on a Likert-type scale ranging from 1 (“Very dissatisfied”) to 5 (“Very satisfied”). Although single-item indicators are less detailed than multi-item instruments, they have been shown to be acceptable proxies of sleep quality in previous studies (Cappelleri et al., 2009; Klimt et al., 2023) and allow for efficient assessment while minimizing participant burden in large samples.

### 2.3. Statistical Analysis

Analyses were conducted on participants who completed the relevant questionnaires. Within each questionnaire, all items were mandatory, so missing values only occurred if a participant discontinued the survey before reaching that section. Participants who did not complete a given questionnaire were excluded from analyses involving that specific measure, resulting in complete datasets for each instrument. Given the cross-sectional design and the completeness of the analytic datasets, imputation or sensitivity analyses for missing data were deemed unnecessary.

Descriptive statistics were computed for all study variables. Continuous variables were tested for normality using the Shapiro–Wilk test. As none of the variables were normally distributed (*p* < 0.001), nonparametric tests were used for group comparisons. Preliminary analyses included Spearman correlations to assess associations between continuous variables, and Kruskal–Wallis and Jonckheere–Terpstra tests to examine group differences across categorical levels of physical activity (low, moderate, high) and sleep quality (poor, average, good), given the non-normal distribution of dependent variables.

Subsequently, linear regression analyses were performed to assess the predictive value of sleep (hours of sleep per night) and physical activity (MET-minutes/week) on four academic well-being outcomes: academic stress, academic self-efficacy, dropout intention, and academic motivation (autonomous and controlled). Gender and age were included as covariates in all regression models. We also examined potential differences across the four fields of study (Health sciences, Engineering and agriculture, Economics, and Law) using Kruskal–Wallis tests. Although significant differences emerged for most outcomes, the patterns were inconsistent across variables, and not all fields significantly differed from each other. Therefore, this variable was not included as a covariate in the regression models.

Prior to conducting regressions, key assumptions were tested. Residual normality was assessed using Q–Q plots and the Kolmogorov–Smirnov and Shapiro–Wilk tests, which indicated deviations from normality (all *p* < 0.001). However, given the large sample size, parametric tests are known to be robust to violations of normality (Blanca et al., 2017; Lumley et al., 2002). Visual inspection of scatterplots of standardized predicted values versus standardized residuals showed no violations of linearity or homoscedasticity. Additionally, multicollinearity diagnostics showed tolerance values close to 1 and Variance Inflation Factors (VIFs) well below the critical threshold, with all values close to 1, indicating no concerns regarding multicollinearity.

For this reason, and considering the robustness of linear models in large samples, linear regressions were deemed appropriate and sufficiently robust despite deviations from normality. All analyses were conducted using IBM SPSS Statistics (version 29).

## 3. Results

In this sample, 41.9% of students scored above the USS cutoff, indicating significant psychological distress related to academic stressors. Approximately 5% of students reported a mean dropout intention score of 4 or higher, indicating frequent or constant thoughts of leaving university. One in five students reported a low level of physical activity, although the overall sample mean exceeded 3000 MET–minutes/week, a value typically associated with high physical activity levels. Notably, one in four students reported 4000 or more MET–minutes/week of physical activity.

Regarding sleep, only about 35% of students reported being quite or very satisfied with their sleep quality, and just 28.9% reported sleeping seven or more hours per night. Detailed questionnaire scores are presented in Table 1. Sociodemographic characteristics by levels of sleep satisfaction and physical activity are presented in Supplementary Table S2.

**Figure 2:**
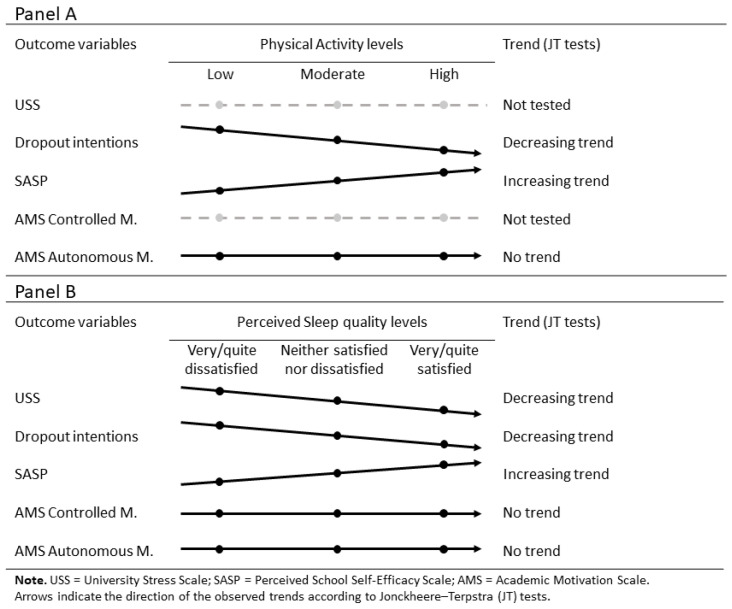
Summary of directional trends across physical activity (Panel **A**) and sleep (Panel **B**) categories.

Table 2 and Figure 2 show the results of the nonparametric tests examining differences and trends in academic outcomes according to sleep satisfaction and physical activity levels. Sleep satisfaction showed significant group differences for USS, dropout intention, SASP, and AMS Controlled and Autonomous Motivation. The Jonckheere–Terpstra test indicated a significant decreasing trend in USS and dropout intention scores, and an increasing trend in SASP scores across higher levels of sleep satisfaction. No significant trend was found for either AMS motivation index.

Physical activity level was significantly associated with dropout intention, SASP, and AMS Autonomous Motivation, showing decreasing trends for dropout intention and increasing trends for SASP scores. Again, no significant trend emerged for AMS Autonomous Motivation scores. Effect sizes for the Kruskal–Wallis tests, expressed as eta squared (η^2^), were generally small, ranging from 0.0028 to 0.0316, indicating modest between-group differences for both physical activity and sleep categories across academic outcomes. Kruskal–Wallis tests indicated significant but non-systematic differences across fields of study (Health sciences, Engineering and agriculture, Economics, and Law) for most outcomes (see Supplementary Materials, Table S3). Specifically, students in Health Sciences reported lower dropout intention and AMS Controlled Motivation scores but higher USS and AMS Autonomous Motivation scores compared with other fields (Economics and Engineering/Agriculture). Law students showed longer sleep duration and higher AMS Autonomous Motivation score than those in Engineering and Agriculture. No significant group differences emerged for SASP score or total physical activity (MET–minutes/week).

Table 3 presents the bivariate Spearman correlations among the study variables. Sleep duration (hours of sleep) was negatively correlated with USS, dropout intention, and academic stress, and positively correlated with SASP and AMS Controlled Motivation. MET–minutes/week showed small negative correlations with dropout intention and AMS Controlled Motivation, and a positive correlation with SASP. All outcome variables were significantly intercorrelated. USS and dropout intention were positively associated with each other and with AMS Controlled Motivation, while both were negatively associated with SASP and AMS Autonomous Motivation. In contrast, SASP and AMS Autonomous Motivation were positively correlated.

Multiple linear regressions were conducted to examine the predictive value of physical activity (MET–minutes/week) and sleep duration on four academic well-being outcomes: USS, dropout intention, SASP, and AMS Controlled Motivation (Table 4). Gender (0 = male, 1 = female) and age were included as control variables in all models. Regression analysis for AMS Autonomous Motivation was not performed, as this variable was not significantly correlated with either sleep duration or physical activity. The models were statistically significant for USS (F (4, 1463) = 25.61, *p* < 0.001), dropout intention (F (4, 1742) = 6.48, *p* < 0.001), SASP (F (4, 1223) = 11.89, *p* < 0.001), and AMS Controlled Motivation (F (4, 1220) = 2.46, *p* = 0.044). Overall, the explained variance was modest across models (R^2^ values ranged from 0.008 to 0.065).

Sleep duration emerged as the most consistent predictor across outcomes, showing significant associations with higher SASP scores and lower USS, AMS Controlled Motivation, and dropout intention scores. Physical activity was positively associated with SASP and negatively associated with dropout intention. Gender was a significant predictor only for academic stress, with female students reporting higher stress levels. Age was positively associated with dropout intention and negatively associated with AMS Controlled Motivation.

## 4. Discussion

The present study aimed to examine the associations between sleep quality, physical activity, and various indicators of academic well-being in a sample of Italian university students from a single university. A high proportion of students reported significant levels of academic distress, consistent with findings from other Italian and international studies (Barbayannis et al., 2022; Dagani et al., 2024; Salazar-Granizo et al., 2024). Regarding health-related behaviors, one in five students reported low levels of physical activity, although the sample’s average MET–minutes/week was very high. This pattern may reflect a subset of students with extremely high activity levels, which is common in self-reported physical activity data (Lee et al., 2011). The proportion of low-activity students is consistent with findings from other European university samples, such as Spain (Rodríguez-Romo et al., 2023). In contrast, inactivity tends to be substantially higher among students in developing countries: multinational studies have shown markedly greater proportions of low activity in low- and middle-income settings (Pengpid et al., 2015; Haase et al., 2004), likely reflecting socioeconomic constraints and more limited access to recreational facilities (Liu, 2022). Concerning sleep, about 40% of students reported being quite or very dissatisfied with their sleep quality, aligning with sleep duration data, as only 29% reported sleeping at least seven hours per night, the minimum recommended for young adults (Hirshkowitz et al., 2015). These values are broadly comparable to those reported in other European university samples (Schlarb et al., 2017; Schmickler et al., 2023). Similarly to physical activity, evidence from multinational reviews indicates that sleep behaviors are shaped by cultural values, country-level wealth, and environmental factors (Jahrami et al., 2020; Peltzer & Pengpid, 2015). Such structural differences contribute to cross-national variability in sleep patterns and should be considered when interpreting these results.

The pattern of correlations among the outcome variables supports considering them as multidimensional indicators of academic well-being, as all variables were significantly interrelated. Specifically, academic stress and dropout intention were positively associated with each other and with controlled motivation, and negatively associated with academic self-efficacy. This suggests that students experiencing higher levels of stress and intentions to leave their studies may feel less capable and more externally driven in their academic engagement. In contrast, academic self-efficacy and autonomous motivation were positively correlated, indicating that students who perceive themselves as competent are more likely to be intrinsically motivated and engaged in their studies. Overall, these correlations underscore the interdependent nature of stress, motivation, self-efficacy, and dropout intention as complementary dimensions of students’ academic well-being.

As hypothesized, better sleep and higher levels of physical activity were generally associated with lower academic stress and dropout intention, as well as with greater academic self-efficacy. However, the expected associations with motivational indices were only partially supported. Given the cross-sectional design, these associations should be interpreted cautiously, as causal relationships cannot be inferred, and the observed effects were modest.

Among the two health-related behaviors assessed, sleep showed the most consistent associations across outcomes. Sleep duration was significantly associated with academic stress, dropout intention, self-efficacy, and controlled motivation, extending previous findings that primarily linked insufficient sleep to poorer academic performance (Curcio et al., 2006; Suardiaz-Muro et al., 2020) and highlighting its broader relevance for academic functioning and well-being. The association between sleep quality and academic outcomes also followed clear linear trends, with better sleep quality linked to lower academic stress, dropout intentions, and higher academic self-efficacy. These findings support previous research (Gaultney, 2016; Hartmann & Prichard, 2018; Noor et al., 2025), suggesting that both sleep quantity and perceived quality may play protective roles in students’ engagement and persistence.

In parallel, physical activity, while less consistently associated with academic outcomes, showed significant relationships with dropout intention and academic self-efficacy. Higher levels of physical activity were linked to lower dropout intentions and greater academic self-efficacy, consistent with prior findings emphasizing the positive contribution of physical activity to students’ academic adjustment (Chang et al., 2021; Jiang et al., 2025; K. Wang et al., 2022). Conversely, the lack of association with academic distress did not support our initial hypothesis and contradicts some earlier studies (Tan et al., 2020). However, recent meta-analytic evidence points to mixed findings in this area (Wunsch et al., 2021), suggesting that the relationship between physical activity and academic distress may be more complex.

Although the R^2^ values observed in the regression models were small, this is not unexpected for behavioral and educational research, where multifactorial influences prevail. Such values indicate that sleep and physical activity explain a modest portion of the variance in academic well-being outcomes. Nonetheless, the consistent direction of associations across models provides meaningful insight into their potential role as distal yet relevant predictors. These modest effect sizes, including eta-squared values from Kruskal–Wallis tests and beta coefficients from regression models, may reflect indirect pathways involving psychosocial mechanisms not assessed in the present study. In particular, the very high mean MET–minutes/week values observed may have attenuated the strength of associations with academic outcomes, possibly due to self-report bias. Alternatively, physical activity might exert its effects on academic well-being through indirect routes, such as enhanced resilience and emotion regulation (Qiu et al., 2025), which, in turn, could buffer academic stress and reduce dropout intention. These interpretations underscore the importance of future longitudinal studies examining potential mediating mechanisms and incorporating objective measures of physical activity. Therefore, despite some inconsistencies and the impossibility of drawing causal conclusions, our findings support the relevance of promoting physical activity as a potentially protective factor in students’ academic well-being.

Contrary to our hypotheses, academic motivation appeared to be only weakly associated with sleep and physical activity. While the AMS Autonomous subscale showed significant differences across physical activity levels and categories of sleep quality in the Kruskal–Wallis tests, these results did not follow a clear trend, and no significant correlations emerged when sleep duration or MET–minutes/week were considered as continuous variables. Controlled motivation was significantly associated with sleep duration in the regression model, indicating that students reporting shorter sleep duration tended to show higher levels of externally driven motivation. However, this relationship was modest, and controlled motivation did not show consistent associations with sleep quality or physical activity indicators. Overall, these findings suggest that academic motivation, at least in its autonomous and controlled dimensions, may not be meaningfully related to sleep and physical activity in this sample. This contrasts with earlier research suggesting that healthier behaviors may foster stronger academic motivation (Van de Casteele et al., 2025; Young-Jones et al., 2022). One possible explanation is that motivational regulation is more strongly influenced by contextual and interpersonal factors (e.g., learning climate, peer support) than by individual health behaviors. Additionally, while health-related behaviors like sleep and physical activity may impact general academic well-being, their effects on specific motivational processes could be more distal, indirect, or moderated by other variables.

Notably, some differences emerged between categorical and continuous indicators of sleep and physical activity. Associations with academic outcomes were generally more evident when using categorical variables (sleep satisfaction and physical activity levels) than with continuous measures (sleep hours and total MET–minutes/week). This discrepancy may reflect the fact that categorical measures capture perceived adequacy or threshold effects (e.g., feeling rested), whereas continuous indicators represent quantitative exposure that may overlook subjective or qualitative aspects. These differences suggest that both approaches provide complementary information and underscore the complexity of how sleep and physical activity relate to academic well-being.

Finally, both gender and age were included as control variables in all models. Age showed two small but significant associations: it was negatively related to controlled motivation and positively related to dropout intention. This suggests that older students may feel slightly less driven by external pressures or obligations but, at the same time, may experience higher levels of academic disengagement or thoughts of leaving university. These findings might reflect the complex interplay between motivational regulation and academic persistence across the student life course. Gender was associated with academic stress, with female students reporting higher levels, consistent with prior research showing greater academic pressure and psychological distress among female students (Noor et al., 2025). Together, these effects highlight the relevance of considering socio-demographic factors when interpreting academic well-being outcomes.

Differences across fields of study also emerged, although they were modest and not systematic. These variations may reflect discipline-specific academic demands and lifestyle patterns, suggesting that contextual factors linked to students’ study environments could partly shape their well-being and motivation.

These findings provide valuable insights for the development of institutional strategies to promote student health and academic well-being. The observed associations between longer and better sleep with lower academic stress, lower dropout intention, and greater self-efficacy suggest that universities should prioritize sleep health as part of their well-being initiatives. Practical actions may include educational campaigns on sleep hygiene, time management, and stress reduction, as well as policies aimed at reducing factors that disrupt sleep, such as excessively early class schedules or clustering of deadlines.

Similarly, the links between higher physical activity levels, lower dropout intentions, and higher self-efficacy highlight the importance of promoting active lifestyles as a resource for academic persistence. Universities could encourage active commuting, integrate short movement breaks during classes, and facilitate access to sports facilities or group-based activities.

Given the high prevalence of academic distress, particularly among female students, institutions should adopt comprehensive and gender-sensitive well-being strategies, integrating psychological support, faculty training, and environmental modifications that foster inclusion and stress prevention. Overall, these findings reinforce the notion that sleep and physical activity are not merely health-related behaviors but integral components of academic health promotion policies.

### 4.1. Limitations

Several limitations should be considered when interpreting the findings of this study. First, all data were based on self-report measures, which may be subject to social desirability bias and recall inaccuracies. In particular, physical activity levels may have been overestimated, as the IPAQ-SF tends to inflate activity estimates due to its reliance on subjective reporting (Lee et al., 2011) compared to accelerometer-based measures. Nevertheless, the IPAQ-SF remains one of the most widely used and validated instruments for assessing physical activity in large populations (Craig et al., 2003; van Poppel et al., 2010), allowing meaningful comparisons with previous research and reliable assessment of relative differences across groups. Moreover, because this study was cross-sectional and primarily aimed to explore associations rather than to estimate absolute activity levels, sensitivity analyses were not conducted.

A further limitation concerns the assessment of sleep, which relied on a single self-report item to evaluate perceived sleep quality. Although this approach does not capture the multidimensional nature of sleep, single-item indicators have been shown to provide acceptable estimates of general sleep quality (Cappelleri et al., 2009; Klimt et al., 2023). Considering the need to minimize participant burden in an online survey that included several standardized instruments, this choice represented a reasonable trade-off between data quality and feasibility.

The cross-sectional design of the study also prevents any conclusions about the causality of the observed associations. An additional potential limitation concerns the moderate response rate: approximately 16% of invited students accessed the survey, and 9% completed all questionnaires. Analyses included slightly different subsets of participants, considering only those who completed the relevant questionnaires. Students who completed the later sections may differ systematically from those who abandoned the survey earlier, which could affect the generalizability of the findings. Future research should consider strategies to increase completion rates or assess differences between completers and non-completers.

Moreover, some potentially relevant variables could not be included in the analyses. For instance, socioeconomic status and year of study were not collected: the latter was excluded due to the heterogeneous duration of academic programs (ranging from three to six years), which made direct comparisons across courses difficult. Furthermore, the sample was drawn from a single Italian university, which may limit the generalizability of the findings to other academic or cultural contexts. Future studies should aim to address these limitations by employing longitudinal or experimental designs, using objective or multimodal assessments, and recruiting more diverse and international samples. Including participants from different countries or cultural backgrounds would allow for the examination of potential cross-cultural differences in the relationships between health-related behaviors and academic well-being.

### 4.2. Practical Implications and Future Directions

Despite these limitations, the present findings suggest that sleep and physical activity may serve as promising entry points for interventions aimed at supporting students’ academic functioning and well-being. Universities could integrate brief, low-cost initiatives (such as workshops on sleep hygiene, group-based physical activity sessions, or online programs) within existing academic support and mental health services. These initiatives may help students build healthier routines, manage academic distress, and strengthen self-efficacy.

However, the modest effect sizes and limited associations with motivation suggest that lifestyle interventions alone may be insufficient to promote deeper psychological or motivational change. Future programs may be most effective when embedded into broader, multidimensional strategies involving university teaching and learning organization, as well as collaboration with student services and local health promotion offices.

From a research perspective, future studies should employ longitudinal and multicentered designs, ideally supported by partnerships between universities and public health institutions, to test causal pathways and ensure generalizability across cultural contexts. The use of objective or multimodal assessments (e.g., actigraphy for sleep and wearable devices for physical activity tracking) would provide more reliable insights into daily behavior patterns. Finally, exploring potential mechanisms and moderators, including resilience, emotion regulation, social support, and learning climate, could clarify how health-related behaviors contribute to students’ academic adjustment and persistence.

## 5. Conclusions

This study examined the associations between sleep and physical activity and academic well-being in a sample of Italian university students. Both sleep and physical activity were linked to key academic outcomes, particularly academic stress, dropout intention, and self-efficacy, whereas associations with academic motivation were less robust. These findings highlight how sleep and physical activity can contribute to students’ psychological and academic adjustment, suggesting that regular physical activity and adequate sleep may foster self-regulatory processes that support effective coping and academic engagement. Although the cross-sectional design and reliance on self-report measures limit causal interpretation, the study offers insight into how everyday behaviors may shape students’ ability to cope with academic demands. Promoting healthy routines within the university context could represent a cost-effective strategy to enhance well-being and reduce dropout risk. Future research and institutional policies should further explore these pathways to support students’ long-term academic success.

## Figures and Tables

**Table 1:** Questionnaires’ scores.

Questionnaire	n	Mean	SD	Scale Range	Shapiro-Wilk *p*
USS	1493	15.02	7.90	0–63	<0.001
SASP	1249	3.09	0.70	1–5	<0.001
Dropout Intention	1775	2.02	0.95	1–5	<0.001
AMS—controlled motivation	1246	6.52	3.96	0–20	<0.001
AMS—autonomous motivation	1246	15.74	3.71	0–20	<0.001
Total MET–minutes/week	1898	3885.98	4091.57		<0.001
Hours of sleep per night	1918	6.88	1.08		<0.001
		%			
Levels of physical activity	1898				
Low		19.4			
Moderate		45.3			
High		35.2			
Sleep Satisfaction	1919				
Very dissatisfied		9.0			
Quite dissatisfied		31.5			
Neither satisfied nor dissatisfied		24.3			
Quite satisfied		29.8			
Very satisfied		5.4			

Note. Abbreviations: USS = University Stress Scale; SASP = Perceived School Self-Efficacy Scale; AMS = Academic Motivation Scale; MET = Metabolic Equivalent of Task; SD = Standard Deviation.

**Table 2:** Nonparametric comparisons of outcome variables by sleep quality and physical activity level (Kruskal–Wallis and Jonckheere–Terpstra tests).

Outcome Variable	Grouping Variable	KW χ^2^ (df)	*p*	η^2^	JT z	*p*
USS	Sleep satisfaction	49.155 (2)	<0.001	0.0316	−6.457	<0.001
	PA level	5.801 (2)	0.055	0.0028		
Dropout intention	Sleep satisfaction	29.371 (2)	<0.001	0.0154	−4.614	<0.001
	PA level	7.570 (2)	0.023	0.0035	−2.259	0.024
SASP	Sleep satisfaction	37.181 (2)	<0.001	0.0282	5.573	<0.001
	PA level	26.488 (2)	<0.001	0.0208	4.439	<0.001
AMS Controlled	Sleep satisfaction	9.463 (2)	0.009	0.0060	−0.976	0.329
	PA level	4.392 (2)	0.111	0.0035		
AMS Autonomous	Sleep satisfaction	7.361 (2)	0.025	0.0043	1.073	0.283
	PA level	6.681 (2)	0.035	0.0044	1.276	0.202

Note. Abbreviations: USS = University Stress Scale; SASP = Perceived School Self-Efficacy Scale; AMS = Academic Motivation Scale; KW = Kruskal–Wallis test; JT = Jonckheere–Terpstra test; df = degrees of freedom; η^2^ = eta squared; PA = Physical Activity. The KW test statistic follows a chi-square distribution. *p*-values are two-tailed and asymptotic.

**Table 3:** Spearman correlations.

Variable	1	2	3	4	5	6	7
1. Hours of sleep	—						
2. MET–minutes/week	−0.084 ***	—					
3. USS	−0.221 ***	−0.034	—				
4. Dropout intention	−0.101 ***	−0.064 **	0.363 ***	—			
5. SASP	0.143 ***	0.151 ***	−0.365 ***	−0.456 ***	—		
6. AMS Controlled Motivation	0.059 *	−0.071 *	0.310 ***	0.263 ***	−0.277 ***	—	
7. AMS Autonomous Motivation	−0.045	0.053	−0.101 ***	−0.380 ***	0.368 ***	−0.117 ***	—

Note. Abbreviations: MET = Metabolic Equivalent of Task; USS = University Stress Scale; SASP = Perceived School Self-Efficacy Scale; AMS = Academic Motivation Scale. * = *p* < 0.05, ** = *p* < 0.01, *** = *p* < 0.001.

**Table 4:** Multiple linear regressions predicting academic well-being variables from sleep, physical activity, and gender.

Dependent Variable	β MET (*p*)	β Sleep (*p*)	β Gender (*p*)	β Age (*p*)	R^2^	F (*p*)
USS	−0.010 (0.697)	−0.207 (<0.001)	−0.137 (<0.001)	0.045 (0.078)	0.065	25.613 (<0.001)
Dropout intention	−0.052 (0.029)	−0.099 (<0.001)	−0.002 (0.940)	0.047 (0.050)	0.015	6.447 (<0.001)
SASP	0.111 (<0.001)	0.166 (<0.001)	−0.027 (0.331)	−0.012 (0.668)	0.037	11.885 (<0.001)
AMS Controlled M.	−0.026 (0.369)	−0.059 (0.042)	−0.001 (0.966)	−0.069 (0.016)	0.008	2.456 (0.004)

Note. Abbreviations: MET = Metabolic Equivalent of Task; USS = University Stress Scale; SASP = Perceived School Self-Efficacy Scale; AMS = Academic Motivation Scale. Multiple linear regressions with MET–minutes/week, hours of sleep, and gender (0 = female, 1 = male) as predictors. Values represent standardized coefficients (β), followed by *p*-values. The model for AMS Autonomous Motivation was not performed, as none of the predictors showed significant correlations with the dependent variable.

## Data Availability

The data supporting the findings of this study are not publicly available due to the inclusion of sensitive or confidential information related to students. Access to the data may be granted upon reasonable request to the corresponding author, subject to ethical and legal constraints.

## Supplementary Materials

The following supporting information can be downloaded at https://www.mdpi.com/article/10.3390/ejihpe16010003/s1, Table S1: STROBE Statement—Checklist of items that should be included in reports of cross-sectional studies; Table S2: Sociodemographic characteristics of participants overall and by categories of sleep satisfaction and physical activity levels; Table S3: Nonparametric comparisons of outcome variables by field of study (Kruskal–Wallis test).

## Abbreviations

The following abbreviations are used in this manuscript:

STROBE	Strengthening the Reporting of Observational Studies in Epidemiology
SD	Standard Deviation
USS	University Stress Scale
AMS	Academic Motivation Scale—adapted version
SASP	Perceived School Self-Efficacy Scale
IPAQ-SF	International Physical Activity Questionnaire—Short Form
MET	Metabolic Equivalent of Task
VIFs	Variance Inflation Factors
KW	Kruskal–Wallis test
JT	Jonckheere–Terpstra test
PA	Physical Activity
