# Supplementary material for: Exploring the Role of Sleep and Physical Activity in Academic Stress, Motivation, Self-Efficacy, and Dropout Intention Among Italian University Students
Source: Eur J Investig Health Psychol Educ. 2025 Dec 22;16(1):3. doi: 10.3390/ejihpe16010003 (PMC12839884; doi:10.3390/ejihpe16010003)
Supplement: Supplementary file 1 [file ejihpe-16-00003-s001.zip › ejihpe-3921339-supplementary.pdf]

Supplementary materials

Table S1. STROBE Statement—Checklist of items that should be included in reports of cross-sectional studies

	Item No	Recommendation	Response
**Title and abstract**	1	(*a*) Indicate the study's design with a commonly used term in the title or the abstract	Done (pg1)
		(*b*) Provide in the abstract an informative and balanced summary of what was done and what was found	Done (pg 1)
**Introduction**			
Background/rationale	2	Explain the scientific background and rationale for the investigation being reported	Done (section 1)
Objectives	3	State specific objectives, including any prespecified hypotheses	Done (section 1)
**Methods**			
Study design	4	Present key elements of study design early in the paper	Done (section 2)
Setting	5	Describe the setting, locations, and relevant dates, including periods of recruitment, exposure, follow-up, and data collection	Done (section 2)
Participants	6	(*a*) Give the eligibility criteria, and the sources and methods of selection of participants	Done (section 2.1)
Variables	7	Clearly define all outcomes, exposures, predictors, potential confounders, and effect modifiers. Give diagnostic criteria, if applicable	Done (section 2)
Data sources/ measurement	8	For each variable of interest, give sources of data and details of methods of assessment (measurement). Describe comparability of assessment methods if there is more than one group	Done (section 2.2)
Bias	9	Describe any efforts to address potential sources of bias	Done (section 2.1, 2.2, 2.3)
Study size	10	Explain how the study size was arrived at	Done (section 2.1)
Quantitative variables	11	Explain how quantitative variables were handled in the analyses. If applicable, describe which groupings were chosen and why	Done (sections 2.2, 2.3)
Statistical methods	12	(*a*) Describe all statistical methods, including those used to control for confounding	Done (section 2.3)
		(*b*) Describe any methods used to examine subgroups and interactions	Done (section 2.3)

		(*c*) Explain how missing data were addressed	Done (section 2.3)
		(*d*) If applicable, describe analytical methods taking account of sampling strategy	Done (section 2.3)
		(*e*) Describe any sensitivity analyses	
**Results**			
Participants	13	(a) Report numbers of individuals at each stage of study—e.g., numbers potentially eligible, examined for eligibility, confirmed eligible, included in the study, completing follow-up, and analysed	Done (section 2.1)
		(b) Give reasons for non-participation at each stage	
		(c) Consider use of a flow diagram	
Descriptive data	14	(a) Give characteristics of study participants (e.g., demographic, clinical, social) and information on exposures and potential confounders	Done (section 3)
		(b) Indicate number of participants for each variable of interest	Done (section 3)
Outcome data	15	Report numbers of outcome events or summary measures	Done (section 3)
Main results	16	(*a*) Give unadjusted estimates and, if applicable, confounder-adjusted estimates and their precision (eg, 95% confidence interval). Make clear which confounders were adjusted for and why they were included	Done (section 3)
		(*b*) Report category boundaries when continuous variables were categorized	
		(*c*) If relevant, consider translating estimates of relative risk into absolute risk for a meaningful time period	
Other analyses	17	Report other analyses done—e.g., analyses of subgroups and interactions, and sensitivity analyses	Done (section 3)
**Discussion**			
Key results	18	Summarise key results with reference to study objectives	Done (section 4)
Limitations	19	Discuss limitations of the study, taking into account sources of potential bias or imprecision. Discuss both direction and magnitude of any potential bias	Done (section 4.1)
Interpretation	20	Give a cautious overall interpretation of results considering objectives, limitations, multiplicity of analyses, results from similar studies, and other relevant evidence	Done (section 4)
Generalisability	21	Discuss the generalisability (external validity) of the study results	Done (section 4)
**Other information**			

Funding	22	Give the source of funding and the role of the funders for the present study and, if applicable, for the original study on which the present article is based	Done

Table S2. Sociodemographic characteristics of participants overall and by categories of sleep satisfaction and physical activity levels.

**Variable**	**Total (%)**	**Sleep satisfaction (%)**			**Physical Activity Levels (%)**		
		Very /quite dissatisfied	*Neither satisfied nor dissatisfied*	*Very / quite satisfied*	*Low*	*Moderate*	*High*
**Gender**							
Female	62.7	61.6	63.3	64.8	68.4	67.2	55.6
Male	37.3	38.4	36.7	35.2	16.7	32.8	44.4
**Citizenship**							
Italian	94.5	95.4	93.1	94.8	92.3	95.2	95.2
From other European countries	0.8	0.5	0.9	0.9	0.8	0.6	0.9
From non-European countries	4.7	4.0	6.1	4.3	6.9	4.2	3.9
**Field of study**							
Health Sciences	46.0	48.0	40.6	47.2	44.2	47.8	44.8
Engineering / Agriculture	28.1	27.2	32.1	26.2	24.5	27.6	30.5
Economics	18.1	17.2	20.4	17.8	20.6	17.7	17.4
Law	7.9	7.6	6.9	8.8	10.6	7.0	7.3
**Study year**							
Bachelor degrees – 1st year	25.3	26.8	30.9	20.0	22.1	24.4	27.9
Bachelor degrees – 2nd year	16.0	15.3	20.0	14.2	14.2	15.5	17.7
Bachelor degrees – 3rd year	18.7	18.1	16.6	20.8	19.5	17.7	19.7
Master degrees – 1st year	6.3	7.0	5.0	6.6	6.2	5.8	7.1
Master degrees – 2nd year	8.2	7.1	9.3	8.5	9.1	8.5	7.3
Single-cycle degrees – 1st year	5.5	4.3	4.8	7.2	6.8	5.9	4.0
Single-cycle degrees – 2nd year	3.9	3.9	2.6	4.8	3.5	4.7	3.2
Single-cycle degrees – 3rd year	3.6	3.5	2.4	4.6	5.0	3.0	3.7
Single-cycle degrees – 4th year	4.4	5.3	2.6	4.8	4.1	5.1	4.0
Single-cycle degrees – 5th year	3.6	3.4	3.8	3.8	4.4	4.0	2.7
Single-cycle degrees – 6th year	4.5	5.3	2.1	4.6	5.0	5.3	2.6
**Mean age (SD)**	22.6 (5.4)	22.9 (5.8)	22.4 (5.5)	22.51(5.1)	23.2 (5.8)	22.3 (4.9)	22.7 (5.7)

Note. Percentages are calculated within each category of sleep satisfaction or physical activity level. Year of study is reported for descriptive purposes only due to the heterogeneity of program lengths across disciplines. Abbreviations: SD = Standard Deviation.

Table S3. Nonparametric comparisons of outcome variables by field of study (Kruskal–Wallis test).

Outcome Variable	KW χ^2(df=3)	*p*	Post hoc summary
USS	18.890	<.001	Health Sciences > Engineering and agriculture
Dropout intention	15.908	<.001	Health Sciences < Engineering and agriculture
SASP	.645	.886	No significant differences
AMS Controlled Motivation	24.457	<.001	Health Sciences < Economics
AMS Autonomous Motivation	58.659	<.001	Health Sciences and Law > Engineering and agriculture and Economics
Sleep duration	11.568	.009	Law > Health Sciences and Engineering and agriculture
MET-minutes/week total	5.210	.157	No significant differences
Age	8.360	0.39	Law > Engineering and agriculture and Economics

Note. Abbreviations: USS = University Stress Scale; SASP = Perceived School Self-Efficacy Scale; AMS = Academic Motivation Scale; MET = Metabolic Equivalent of Task. KW = Kruskal–Wallis test; df= degrees of freedom. The KW test statistic follows a chi-square distribution. *p*-values are two-tailed and asymptotic. The post hoc summary column indicates significant pairwise differences between groups, with “>” denoting that the first group had higher values than the second, and “<” denoting that the first group had lower values than the second. Post hoc comparisons were adjusted using the Bonferroni correction.
